# Validation of T1 and T2 algorithms for quantitative MRI: performance by a vendor-independent software

**DOI:** 10.1186/s12880-016-0148-6

**Published:** 2016-08-08

**Authors:** Sebastian Bidhult, George Kantasis, Anthony H. Aletras, Håkan Arheden, Einar Heiberg, Erik Hedström

**Affiliations:** 1 Department of Clinical Sciences Lund, Clinical Physiology, Lund University, Skane University Hospital, Lund, Sweden; 2 Department of Biomedical Engineering, Faculty of Engineering, Lund University, Lund, Sweden; 3 Laboratory of Medical Informatics, School of Medicine, Aristotle University of Thessaloniki, Thessaloniki, Greece; 4 Department of Clinical Sciences Lund, Diagnostic Radiology, Lund University, Skane University Hospital, Lund, Sweden

**Keywords:** T1, T2, Mapping, Quantitative magnetic resonance imaging

## Abstract

**Background:**

Determination of the relaxation time constants T1 and T2 with quantitative magnetic resonance imaging is increasingly used for both research and clinical practice. Recently, groups have been formed within the Society of Cardiovascular Magnetic Resonance to address issues with relaxometry. However, so far they have avoided specific recommendations on methodology due to lack of consensus and current evolving research. Standardised widely available software may simplify this process.

The purpose of the current study was to develop and validate vendor-independent T1 and T2 mapping modules and implement those in the versatile and widespread software Segment, freely available for research and FDA approved for clinical applications.

**Results:**

The T1 and T2 mapping modules were developed and validated in phantoms at 1.5 T and 3 T with reference standard values calculated from reference pulse sequences using the Nelder-Mead Simplex optimisation method. The proposed modules support current commonly available MRI pulse sequences and both 2- and 3-parameter curve fitting. Images acquired in patients using three major vendors showed vendor-independence. Bias and variability showed high agreement with T1 and T2 reference standards for T1 (range 214–1752 ms) and T2 (range 45–338 ms), respectively.

**Conclusions:**

The developed and validated T1 and T2 mapping and quantification modules generated relaxation maps from current commonly used MRI sequences and multiple signal models. Patient applications showed usability for three major vendors.

## Background

Quantitative magnetic resonance imaging (MRI) is increasingly used for several different applications in both research and clinical practice. For cardiac MRI, T1 quantification enables measurement of myocardial extracellular volume [1–3], whereas T2 mapping detects oedema in acute myocardial infarction [4]. The Society of Cardiovascular Magnetic Resonance (SCMR) has also recently formed groups and provides general recommendations on use of mapping for research and clinical applications [5, 6]. However, specific recommendations have been avoided so far due to lack of consensus and current evolving research [6]. The Society is thus awaiting this field to develop so that guidelines can be properly formulated. Standardised software may simplify and speed up this process.

Further, cancer imaging has benefited from T1 to T2 mapping for determining early tumour progression in brain [7], and provides improved discrimination between benign and malign findings in suspected prostate cancer [8]. Also, oxygen saturation in blood has been accurately measured noninvasively by T2 mapping in children with complex congenital heart disease, yielding an opportunity to potentially avoid cardiac catheterisation for follow-up studies in children [9].

Different numerical algorithms can be used to compute T1 and T2 relaxation maps and therefore inline map generation may vary between MRI vendors. Also, most inline systems do not present the curve fit, which, if visualised, can be used as a marker of accuracy. Moreover, the algorithms used are not openly documented. A previous open-source software overcame these limitations, but is only to be used for research [10]. Last but not least, current available software is generally limited in signal models and fitting options.

The purpose of this study was to develop, validate and openly document T1 and T2 relaxation map modules with multiple signal models, test those in images acquired using three major vendors, and implement the validated modules in freely available software for research [11].

## Implementation

The T1 and T2 mapping modules were developed and validated in phantoms with reference standard T1 and T2 values calculated from reference spin echo (SE) pulse sequences using the Nelder-Mead Simplex optimisation method available in Matlab (Math Works, Natick, MA; 2014a). The proposed modules support current commonly available MRI pulse sequences and both 2- and 3-parameter curve fitting (Table 1).

**Table 1:** Supported sequences and signal fitting models

	Supported signal models	
Supported sequences	3-parameter fit model	2-parameter fit model
T1 spin-echo IR (magnitude images)	S(t) = |A (1 − B exp(−t/T1))|	S(t) = |A (1 − 2 exp(−t/T1))|
T1 PSIR	S(t) = A (1 − B exp(−t/T1))	S(t) = A (1 − 2 exp(−t/T1))
T1 saturation recovery balanced SSFP	S(t) = A (1 − B exp(−t/T1))	S(t) = A (1 − exp(−t/T1))
T1 MOLLI/T1 Look-Locker correction (magnitude images)	S(t) = |A (1 − B exp(−t/T1*))|; T1 = T1* (B − 1)	n/a
T1 MOLLI/T1 Look-Locker correction (PSIR images)	S(t) = A (1 − B exp(−t/T1*)); T1 = T1* (B − 1)	n/a
T2 spin echo (multi-echo and single-echo)	S(t) = A exp(−t/T2) + B; B > 0	S(t) = A exp(−t/T2)
T2-prepared balanced SSFP	S(t) = A exp(−t/T2) + B; B > 0	S(t) = A exp(−t/T2)

*IR* inversion recovery, *PSIR* phase sensitive inversion recovery, *MOLLI* modified Look-Locker inversion recovery, *SSFP* steady-state free precession

**Fig. 1:**
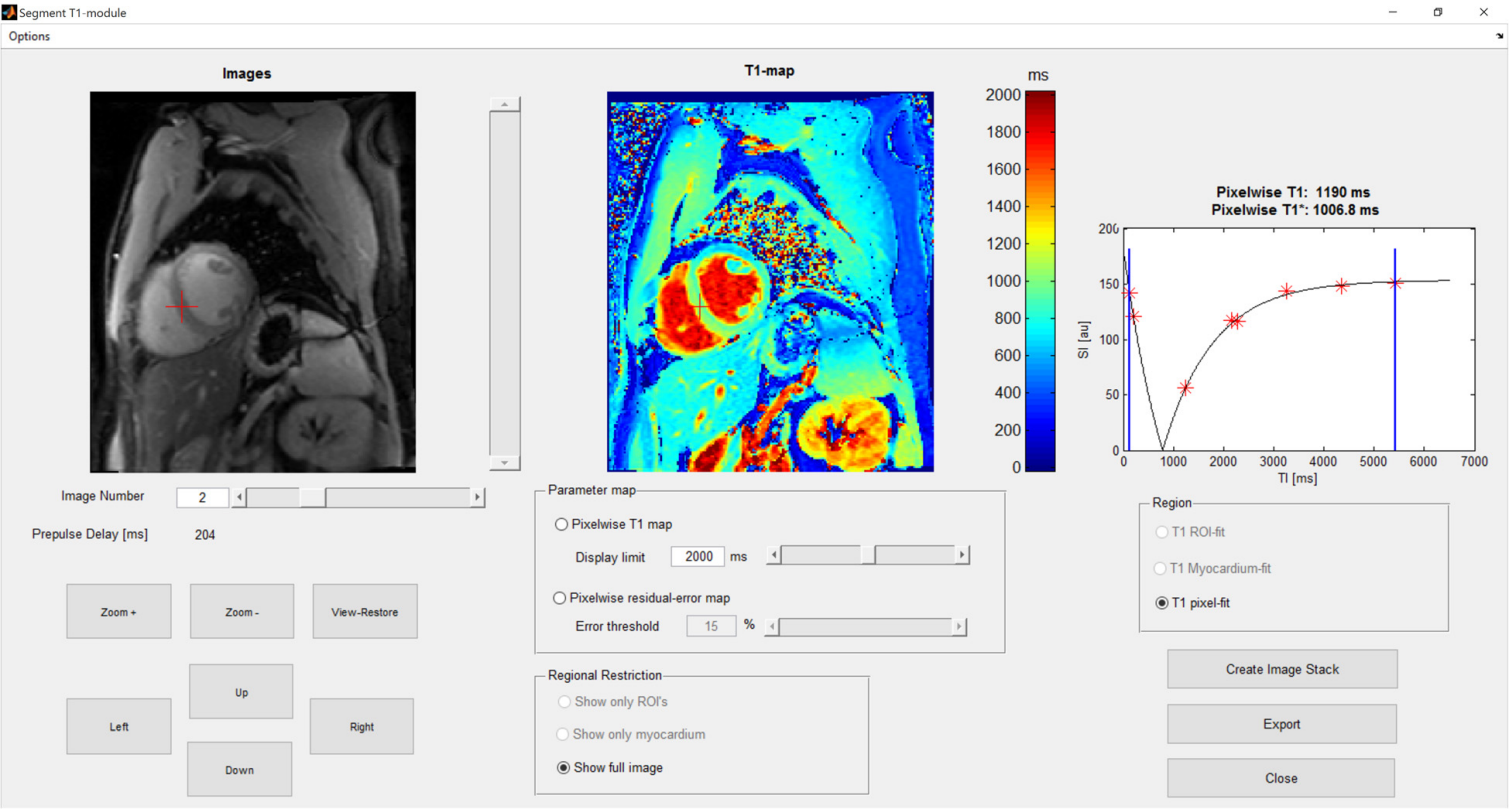
The image shows the graphical user interface of the T1 mapping module (the T2 mapping module shares the design). An example of a cardiac pre-contrast MOLLI T1 map at 1.5 T in a healthy volunteer is shown. Both pixel-wise mapping, for the whole image and ROI-based, and ROI-based global mean mapping are available. The residuals of the curve fit for both T1 and T2 mapping can be visualised, indicating areas within the image that have a higher deviation from the curve and thus less accurate T1 and T2 values

The T1 mapping module graphical user interface is shown in Fig. 1 (the T2 mapping module shares the design). Both ROI-based global mean mapping and pixel-wise mapping for the whole image and ROI-based are available. Further, the residuals of the curve fit for both T1 and T2 mapping can be visualised, indicating areas within the image that have a higher deviation from the curve and thus less accurate T1 and T2 values.

The validated modules were finally implemented in the software Segment, freely available for research (http://www.medviso.com) [11].

Since Segment already includes a validated module for T2* mapping and quantification [12], this topic was not covered in the current study.

### Phantom setup and imaging

A Eurospin (Diagnostic Sonar, Livingston, UK) phantom encompassing 12 gadolinium/agarose gel phantoms was used for validation of the proposed modules. The phantoms were scanned at both 1.5 T and 3 T (Siemens Aera and Prisma, Erlangen, Germany). Single-echo spin-echo sequences were used for acquiring the reference T1 and T2 values. Pulse sequence parameters are presented in Table 2. The magnetization was allowed to fully recover between spin radiofrequency excitations.

**Table 2:** Typical MRI sequence parameters

	TE (ms)	TR (ms)/delay between contrast preparations pulses	FA (°)	FOV (mm)	Matrix	Preparation pulse delays (ms)	iPAT/SENSE factor	Receiver BW (kHz)	ACQ time (hh:mm:ss)
T1 spin-echo IR (magnitude images)	5.8	10,000/10,000	90	241 × 241	128 × 128	[21, 60, 100, 200, 300, 500, 660, 900, 1050, 1300, 1600, 2000, 2250, 2500, 3000, 3500, 4300]	off	64	06:45:20
T1 PSIR	1.11	2.4/40,000	35	360 × 270	192 × 144	[150, 300, 400, 500, 700, 900, 1100, 1300, 1800, 2000, 2500, 3000, 3700, 4300, 5000, 6300] + 1 reference image without IR-preparation	2	208.32	00:11:20
T1 saturation recovery balanced SSFP	1.11	2.4/40,000	35	360 × 270	192 × 144	[150, 300, 400, 500, 700, 900, 1100, 1300, 1800, 2000, 2500, 3000, 3700, 4300, 5000, 5600, 6300, 8000] + 1 reference image without SR preparation	2	208.32	00:12:40
T1 MOLLI/T1 Look-Locker correction (magnitude and PSIR images)	1.1	2.4/7920	35	360 × 270	192 × 144	[130, 210, 1130, 1210, 2130, 2210, 3130, 4130]	2	208.32	00:00:11
T2 spin echo (single-echo)	[6, 12, 20, 30, 40, 50, 70, 90, 120, 140, 180, 300, 400, 600, 1000]	10,000/10,000	90	241 × 241	128 × 128	n/a	off	64	05:20:00
T2 spin echo (multi-echo)	[9, 18, 27, 36, 45, 54, 63, 72, 81, 90, 99, 108, 117, 126, 135, 144]	1500/n/a	90	160 × 160	256 × 256	n/a	off	62.5	00:06:24
T2-prepared balanced SSFP	1.11	2.4/20,000	35	360 × 270	192 × 144	T2p range = 25 − 200; ΔT2p = 5;	2	208.32	00:12:20

*IR* inversion recovery, *PSIR* phase sensitive inversion recovery, *MOLLI* modified Look-Locker inversion recovery, *SSFP* steady-state free precession

Commonly available T1 and T2 mapping sequences were used to acquire images of the phantom, and the proposed modules were applied to generate T1 and T2 relaxation constant maps. The sequences were based on a free-breathing single-shot balanced steady-state free precession (bSSFP) sequence.

### T1 mapping

Pixelwise T1 estimates were initialized using a lookup-table search performed in two steps in a T1 interval of 0–4000 ms. First, a step of 50 ms between lookup-table entries was applied for high performance. Thereafter, to find the optimal value, a second search was performed using a 5 ms difference between lookup-table entries within 100 ms of the entry found in the first step. In these two steps, depending on the pulse sequence, ideal inversion/saturation efficiency was assumed and each pixel was normalized with the maximum absolute value within its time-series. The T1 lookup-table entry resulting in the minimum sum of absolute error was chosen as the initial T1 value.

Following T1 initialization, pixel T1 values were refined using a C implementation of the Nelder-Mead Simplex nonlinear optimisation algorithm [13]. Convergence was assumed when the maximum T1 absolute difference between two simplexes was less than 0.10 ms. The C implementation was performed to reduce computation times and was thus not used for calculating the ROI-based global mean where instead the pre-implemented Matlab fminsearch method [14] was sufficient.

For T1 reference values, an inversion recovery (IR) single-echo spin echo sequence was used with a short echo time and long repetition time (Table 2). Two variations of the free-breathing bSSFP sequence were used for T1 mapping; one based on SR and one based on IR preparation pulses respectively applied before imaging readout. At 1.5 T, T1 was also estimated using a breath-hold MOLLI sequence with pre- and post-contrast cardiac configurations (5(3b)3 and 4(1b)3(1b)2) for analysis of phantoms with T1 > 600 ms and T1 < 600 ms, respectively. Since the MOLLI acquisition alters the recovery curve in itself, inducing T1* measurements, the Look-Locker correction from T1* to T1 was performed (Table 1), as previously proposed [15]. Magnitude images were used to estimate T1 from spin echo, SR-bSSFP and MOLLI sequences. For IR-bSSFP, the phase and magnitude images were extracted in order to reconstruct phase-sensitive inversion recovery (PSIR) images, as previously proposed [16].

### T2 mapping

The initial T2 estimate was initialized by a weighted 2-parameter linear regression of the signal logarithm [17]. The estimation was repeated for stepwise truncation of the maximum echo time until three data points remained. The T2 estimate resulting in the minimum sum of absolute error over all data points was chosen as the initial T2 estimate.

Following the T2 initialisation, all pixels with T2 values outside the interval 0 < T2 < 400 ms were excluded from further analysis and the final T2 estimate was refined using the above-mentioned nonlinear optimisation algorithm. Convergence was assumed when the maximum T2 absolute difference between two simplexes was less than 0.10 ms. Pixels that were not refined with nonlinear optimization were set to 0 in the resulting T2 map.

For T2 reference values, a single-echo spin echo sequence was used. The free-breathing bSSFP sequence used T2 preparation pulses for T2 mapping. An SR-prepared image with a short saturation time was used for the T2 calculation in order to improve the 3-parameter curve fit [18]. Magnitude images were used to estimate T2 from both spin echo and bSSFP sequences.

### Residual calculation for T1 and T2 mapping

Curve-fit residuals for T1 and T2 mapping were calculated as the average absolute difference between the fitted curve and corresponding pixel values. Residuals were normalised relative to the maximum absolute pixel value within its time-series and reported as a percentage.

### Application on human MR images

The developed and validated T1 and T2 mapping modules were applied on images acquired from three major vendors. Standard available sequences were used on Siemens (1.5 T Aera and 3 T Prisma, Erlangen, Germany) with 60-channel phased array coils and a 20-channel head coil; Philips (1.5 T Achieva, Best, the Netherlands) with 32-channel phased array coils; and on GE (3 T Discovery 750w, General Electrics, USA) with a GEM flex medium array coil. The local ethics committee approved the research protocol and all subjects provided written consent.

### Statistics

Bias and variability were determined using the modified Bland-Altman analysis. The bias and variability percentages were computed as the difference between the proposed method and the reference standard divided with the reference standard values. Values were expressed as mean ± SD and 95 % limits of agreement.

## Results

Computational times were generally fast independent of amount of information, i.e. for both full image and ROI-based calculations (Table 3).

**Table 3:** Computational times for pixel-wise mapping in the complete image and in a selected ROI, respectively

	Pixel-wise (complete image)	Pixel-wise (ROI only)
T1 spin-echo IR (magnitude images)	2.5 s (3 parameters, 17 images, 128 × 128 images, 13 435 refined pixels)	0.03 s (3 parameters, 17 images, 200 pixels, 152 refined pixels)
T1 PSIR	11.7 s (3 parameters, 17 images, 192 × 144 matrix, 26 597 refined pixels)	0.15 s (3 parameters, 17 images, 200 pixels, 200 refined pixels)
T1 saturation recovery balanced SSFP	11.2 s (3 parameters, 15 images, 192 × 144 matrix, 26 594 refined pixels)	0.09 s (3 parameters, 15 images, 202 pixels, 202 refined pixels)
T1 MOLLI/T1 Look-Locker correction (magnitude and PSIR images)	6.4 s (3 parameters, 9 images, 192 × 144 matrix, 25 128 refined pixels)	0.05 s (3 parameters, 9 images, 202 pixels, 198 refined pixels)
T2 spin echo (single-echo and multi-echo)	1.12 s (2 parameters, 15 images, 128 × 128 matrix, 9 175 refined pixels)	0.04 s (2 parameters, 15 images, 207 pixels, 160 refined pixels)
T2-prepared balanced SSFP	11.7 s (3 parameters, 37 images, 192 × 144 matrix, 16 322 refined pixels)	0.14 s (3 parameters, 37 images, 207 pixels, 184 refined pixels)

The ROI-based global mean fitting takes less than 2 s for all sequences and is not listed. Performance was tested at a 2.4 GHz, 8 GB RAM, SSD HDD standard laptop running the MS Windows 7 64-bit operating system

*IR* inversion recovery, *PSIR* phase sensitive inversion recovery, *MOLLI* modified Look-Locker inversion recovery, *SSFP* steady-state free precession

**Fig. 2:**
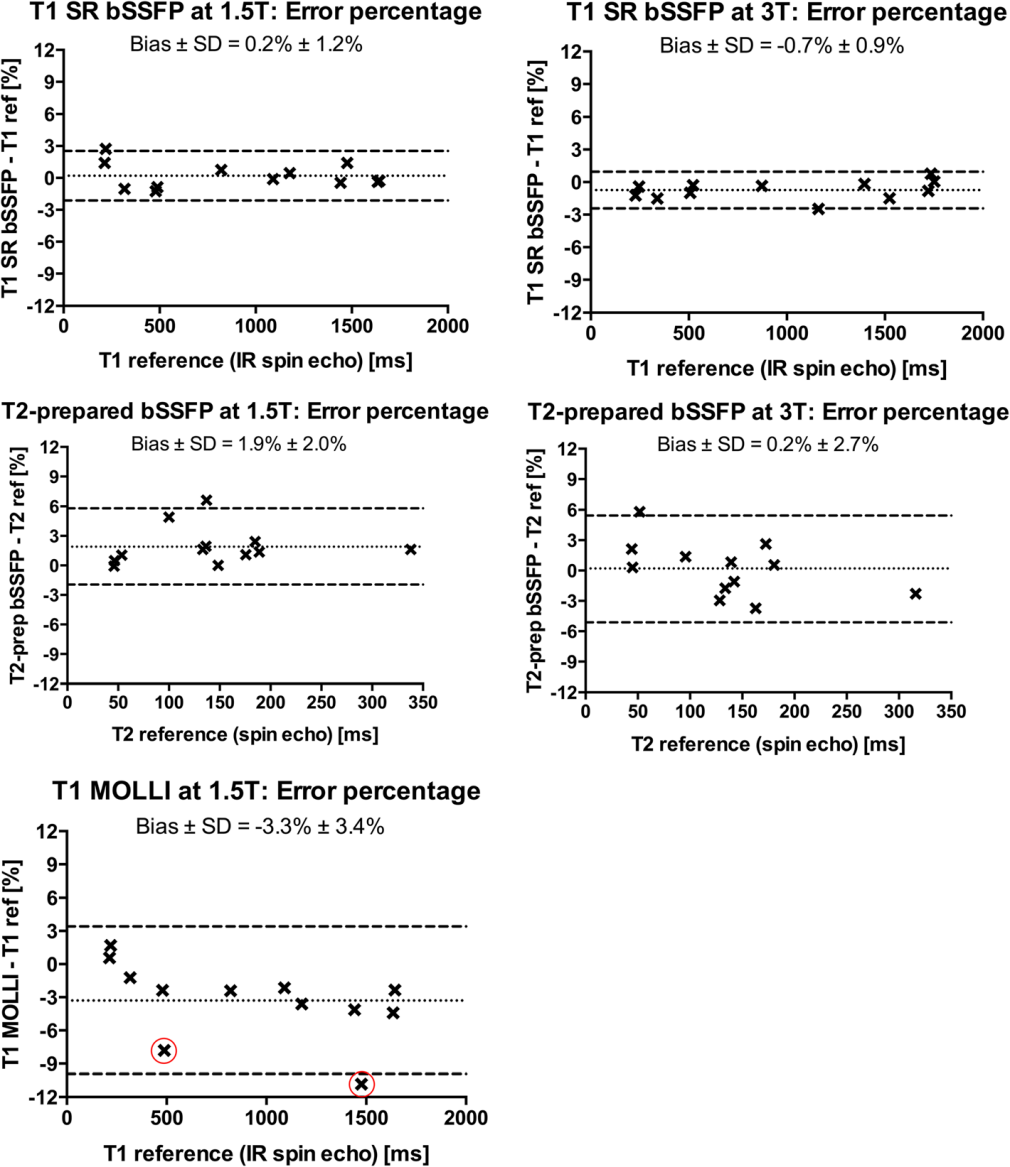
Modified Bland-Altman analyses of the phantom validation data at 1.5 T (*left column*) and 3 T (*right column*). All curve fits were performed using the 3-parameter fit. The dotted and dashed lines represent bias and 95 % limits of agreement. A generally high agreement was found. The T1 outliers dependent on low T2 (<60 ms) found using the MOLLI sequence at 1.5 T are encircled in *red*. bSSFP = balanced steady-state free precession; MOLLI = modified Look-Locker inversion recovery; SR = saturation recovery; IR = inversion recovery

**Fig. 3:**
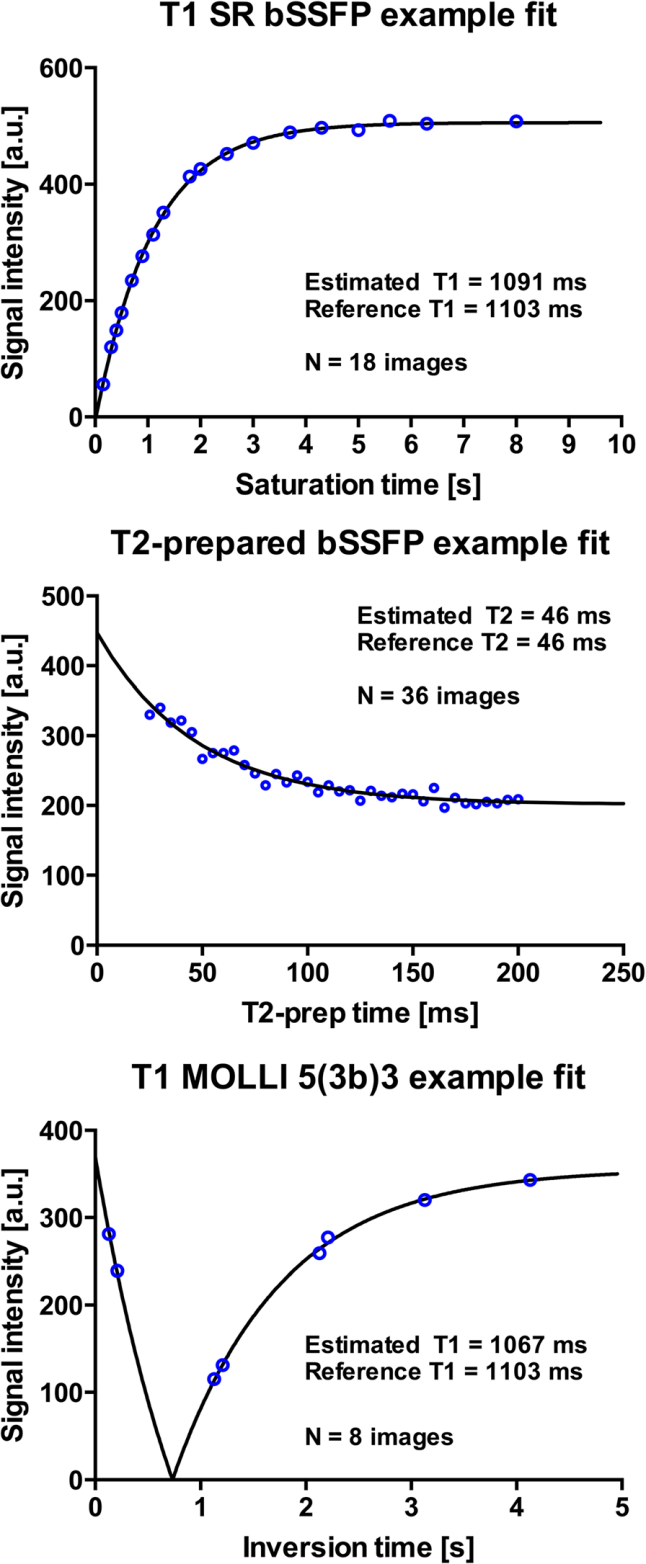
Example curve fits for T1 and T2 at 1.5 T in two phantoms. The solid lines represent estimated relaxation curves. bSSFP = balanced steady-state free precession; MOLLI = modified Look-Locker inversion recovery; SR = saturation recovery

The T1 and T2 reference values ranged from 214 to 1643 ms and 46–338 ms for 1.5 T, and 229–1752 ms and 45–316 ms for 3 T, respectively. Phantom validation results are shown for 1.5 T and 3 T (Fig. 2) and corresponding curve fit examples at 1.5 T (Fig. 3). The T1 bias and variability at 1.5 T were 0.8 ± 8 ms (0.2 ± 1.2 %) for SR-bSSFP using the 3-parameter fit, and 24 ± 9 ms (3.5 ± 2.3 %) using the 2-parameter fit. Corresponding bias and variability for PSIR-bSSFP at 1.5 T were 3.2 ± 3.8 ms (0.6 ± 1.0 %) and −31 ± 26 ms (−3.5 ± 2.1 %). For cardiac MOLLI at 1.5 T the bias and variability was −39 ± 45 ms (−3.3 ± 3.4 %). The higher variability for MOLLI was related to low T2, with errors above 5 % originating from phantoms with reference T2 values < 60 ms.

The T1 bias and variability at 3 T were for SR-bSSFP −6 ± 11 ms (−0.7 ± 0.9 %) when applying a 3-parameter fit, whereas a 2-parameter fit yielded 22 ± 13 ms (2.8 ± 1.6 %). Corresponding bias and variability for PSIR-bSSFP at 3 T were −9 ± 13 ms (−1.1 ± 1.0 %) and −36 ± 33 ms (−3.3 ± 2.1 %).

The T2 bias and variability were 2.8 ± 2.7 ms (1.9 ± 2.0 %) at 1.5 T and −0.7 ± 3.6 ms (0.2 ± 2.7 %) at 3 T using the 3-parameter fit.

**Fig. 4:**
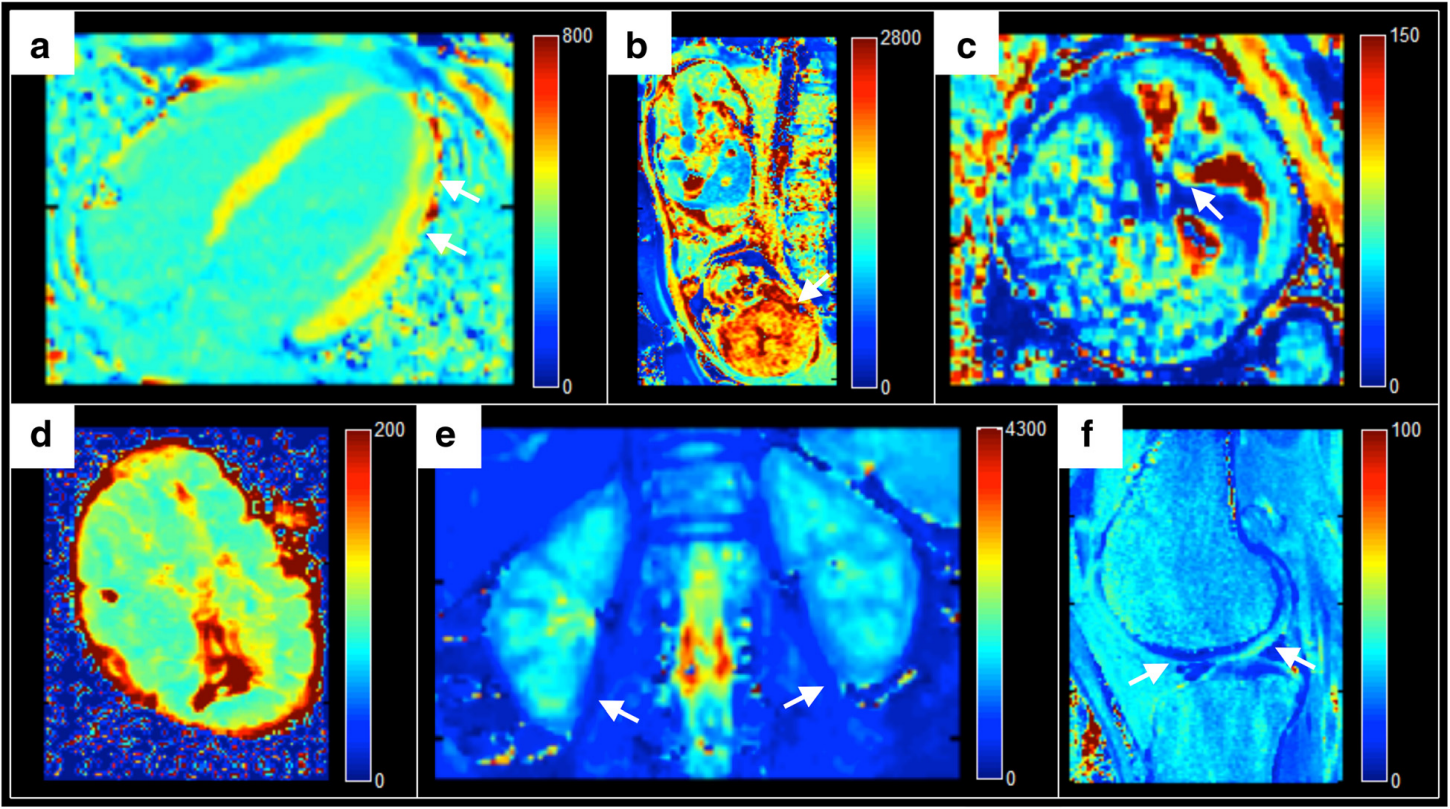
Application of the proposed T1 and T2 mapping modules on images acquired in humans and ex vivo. Data from 3 major vendors were used for reconstruction of T1 and T2 maps. Arrows point to findings or structures. **a** Siemens Aera 1.5 T myocarditis (T1, MOLLI); **b** Siemens Aera 1.5 T healthy foetal brain (T1, inversion-recovery bSSFP); **c** Siemens Aera 1.5 T healthy foetal blood oxygenation in descending aorta (T2, T2-prepared bSSFP); **d** Siemens Prism 3 T ex vivo healthy placenta (T1, inversion-recovery bSSFP); **e** Philips 1.5 T healthy kidney (T1, MOLLI); **f** GE 750w 3 T healthy knee cartilage (T2, multi-echo spin-echo)

Figure 4 shows T1 and T2 maps from human applications for three major vendors.

## Discussion

The developed and validated T1 and T2 mapping modules generated maps from commonly used MRI sequences and multiple signal models. Generally low bias and variability were found compared with reference standard measurements in phantoms. Patient applications showed usability for three major vendors. The main software is freely available for research and well documented.

The proposed algorithms showed particularly good agreement with the reference standard for saturation recovery sequences. However, T1 was underestimated by MOLLI when also phantoms with low T2 values (<60 ms) were included. This is similar to previously published data showing T2 sensitivity for MOLLI T1 mapping, with approximately 5 % error in T1 values for T2 below 30 ms [19]. This underestimation may be corrected for by using recently proposed lookup-table methods [20]. Another explanation for the increased variability using MOLLI compared to saturation recovery may be the reduced number of sampling points used for MOLLI in this study.

The slightly higher, albeit not large, variability shown for the T2 prepared sequence may be explained by limited signal-to-noise ratio. The T2 prepared mapping may be improved by acquiring several data points (echoes), especially late TE images since an offset is known to occur. Another solution is to acquire fewer echoes and instead add a saturation recovery acquisition, as performed in the current study [18]. This solution only adds a single heartbeat to the acquisition time (approximately 1 s) and is therefore applicable in most patients. Preferably, more than 1 saturation recovery acquisition should be added for averaging. This number may on the other hand need to be optimised in the individual case, especially in cardiac disease where the patient may have difficulties extending the duration of the breath hold.

The proposed software modules include both 2- and 3-parameter T1 and T2 fitting. In theory, a reduced number of parameters should result in reduced random errors (i.e. reduced variability) while however also leading to a risk of introducing a bias due to increased sensitivity to measurement imperfections. In cardiac MRI, 3-parameter T1 curve fitting is commonly applied to reduce bias associated with imperfect preparation-pulse efficiency and/or effects from applied readout pulses [21, 22]. The 3-parameter curve fit has also been suggested for cardiac T2 mapping when using the T2-prepared bSSFP sequence [18]. Unbiased 2-parameter fitting for cardiac T1 mapping has recently been proposed [23], which may lead to an increased need for software supporting 2-parameter T1 mapping in the near future. Clinical validation was not performed as part of the current study, as it is important to first validate algorithms that are to be applied in future in vivo studies. Finally, albeit data from three major vendors were tested, other vendors may use other sequences or reporting of data for fitting, and future studies may benefit from including additional vendors.

## Limitations

In the current modules neither rigid nor non-rigid registration has been implemented. These methods may in some cases improve the diagnostic quality and are subject to future improvements and investigations and will be included in future updates of the modules. Further, some MRI vendors use private dicom headers for data needed for T1 and T2 mapping. These headers may change between MRI vendor software updates. Current known private dicom headers have been implemented in the proposed modules and future updates aim to cover these changes.

## Conclusions

The developed and validated T1 and T2 mapping modules generated relaxation maps from current commonly used MRI sequences and multiple signal models. Patient applications showed usability for three major vendors. The main software is freely available for research and well documented.

## Abbreviations

bSSFP, balanced steady-state free precession; IR, inversion recovery; MOLLI, modified Look-Locker inversion recovery; MRI, magnetic resonance imaging; PSIR, phase-sensitive inversion recovery; SCMR, society of cardiovascular magnetic resonance; SE, spin echo; SR, saturation recovery.
